# Do Patients with Diabetes Mellitus and Polytrauma Continue to Have Worse Outcomes?

**DOI:** 10.3390/jcm12103423

**Published:** 2023-05-12

**Authors:** James Tebby, Vasileios P. Giannoudis, Sophia M. Wakefield, Fiona Lecky, Omar Bouamra, Peter V. Giannoudis

**Affiliations:** 1 Academic Department of Trauma & Orthopaedics, School of Medicine, University of Leeds, Leeds LS2 9LU, UKvasileios.giannoudis@doctors.org.uk (V.P.G.); sophwakey@gmail.com (S.M.W.); 2 Trauma Audit and Research Network, Summerfield House, Salford Royal NHS Foundation Trust, Salford M6 8HD, UK or f.e.lecky@sheffield.ac.uk (F.L.); omar.bouamra@tarn.ac.uk (O.B.); 3 Centre for Urgent and Emergency Care Research, School of Health and Related Research (ScHARR), University of Sheffield, Sheffield S1 4DA, UK; 4 NIHR Leeds Biomedical Research Centre, Chapel Allerton Hospital, Leeds LS2 9LU, UK

**Keywords:** polytrauma, multiple injuries, diabetes mellitus, complications, mortality

## Abstract

The management of patients with multiple injuries remains challenging. Patients presenting with comorbidities, such as diabetes mellitus, may have additional unpredictable outcomes with increased mortality. Therefore, we aim to investigate the impact of major trauma centres in the UK on the outcomes of polytrauma patients with diabetes. The Trauma Audit and Research Network was used to identify polytrauma patients presenting to centres in England and Wales between 2012 and 2019. In total, 32,345 patients were thereby included and divided into three groups: 2271 with diabetes, 16,319 with comorbidities other than diabetes and 13,755 who had no comorbidities. Despite an overall increase in diabetic prevalence compared to previously published data, mortality was reduced in all groups, but diabetic patient mortality remained higher than in the other groups. Interestingly, increasing Injury Severity Score (ISS) and age were associated with increasing mortality, whereas the presence of diabetes, even when taking into consideration age, ISS and Glasgow Coma Score, led to an increase in the prediction of mortality with an odds ratio of 1.36 (*p* < 0.0001). The prevalence of diabetes mellitus in polytrauma patients has increased, and diabetes remains an independent risk factor for mortality following polytrauma.

## 1. Introduction

Patients with multiple injuries continue to represent the ultimate challenge to trauma surgeons and trauma care systems. The presence of polytrauma in different body regions and body systems necessitates timely decisions with the involvement of different disciplines. Patients usually present in critical condition with an increased risk of morbidity and mortality [1].

Polytrauma remains the most frequent cause of mortality in young adults, and for this reason, great efforts have been undertaken over the years from different nations around the globe to develop trauma care systems in order to improve the management and outcomes in this cohort of patients [2].

In 2014, our group, using data between 2003 and 2011 from the Trauma, Audit and Research Network (TARN), reported poorer outcomes for patients that suffered from polytrauma and had diabetes mellitus [3]. The data at that time showed statistically higher mortality for diabetic patients (32.4%), especially those who had head injuries (43.9%) [3]. The study also showed that diabetic patients were more likely to sustain polytrauma from a low-height fall compared to a previously healthy cohort (18.5% vs. 2.3%), *p* < 0.05. Diabetic patients also spent more time in hospital [3]. In addition, we were able to show trends towards increased mortality from chest injuries and from any-cause complications and a greater likelihood of developing infective complications, as well as renal failure. The original cohort of diabetic patients numbered just 222 and, therefore, none of these trends were shown to have statistically significant differences compared to the other cohorts.

Established in 1990, TARN is the largest European Trauma registry. It collects anonymised data for major trauma patients from 220 hospitals across England, Wales, Northern Ireland (NI) and the Republic of Ireland (ROI). TARN helped to provide data that were used in the National Confidential Enquiry into Patient Outcome and Death (NCEPOD) 2007 report “Trauma, Who cares?” [4] which led, in part, to the National Audit Office recommending the development of a major trauma network. This network came into effect outside of London in April 2012 [5].

Both trauma and diabetes are increasing factors in global healthcare, with predictions that by 2030, the leading causes of traumatic death and injury (road traffic collisions, murder and suicide) will increase substantially [6], and that up to 5.5 million people will be living with diabetes [7]. The NHS spends at least GBP 10 billion a year on diabetes, which is about 10% of its entire budget [7].

The aim of this study was to use data from the TARN network since the introduction of the major trauma network to look at whether the outcomes for diabetic patients suffering polytrauma have been improved since its introduction and whether diabetic patients continue to have poorer outcomes compared to other cohorts.

## 2. Materials and Methods

### 2.1. Data Sources

TARN data from 2012 to 2019 were searched for patients that suffered from polytrauma (patients from the ROI and NI were excluded as they started submitting data to TARN after 2013 and 2017, respectively). Polytrauma was once again defined as “having suffered an Abbreviated Injury Score (AIS) of 3 or more in any 2 or more body regions (AIS 3+ ≥ 2)” [8]. Complete datasets that provided a primary outcome (survival vs. non-survival) and had a complete recording of the patient’s pre-morbid health status were included. The remaining patients were then split into 3 cohorts: patients with diabetes, patients with previous comorbidities but not diabetes and a third cohort with no diabetes or previous comorbidities. Each set of data was then compared, looking at a primary outcome of mortality and then secondary outcomes, including complications, lengths of stay, mechanism of injury and body area injured. Where applicable, we investigated the changes in outcomes across all groups compared to the original dataset.

### 2.2. Statistical Analysis

Descriptive statistics were used to compare the demographic features, injury characteristics and crude mortality. Excluded patients with unknown final outcomes were used in a sensitivity analysis for selection bias.

For the continuous variables, hypothesis testing between subgroups was performed using K-sample equality-of-median test (Mood’s test). The null K samples were drawn from populations with the same median. This was followed by a post hoc analysis using Dunn’s test for pairwise comparison and using Bonferroni adjustment for multiple comparisons. For the categorical variables, Chi-squared and Fisher’s exact tests were used. Multiple logistic regression was utilised to predict mortality using age, gender and their interactions, Injury Severity Score (ISS), Glasgow Coma Score (GCS) and the exposure factor previous medical comorbidities (PMC) with 3 levels (No PMC, PMC with no diabetes, Diabetes). Missing values were present for GCS (4% missing); therefore, imputation based on missing at random (MAR) assumption was used, with the “mi” procedure with a chained equation with predicted mean matching (pmm). The outcomes and variables used in the logistic regression were used in the imputation model. A two-sided *p*-value of 0.05 was considered to be statistically significant. Statistical analysis was performed using the Stata software version 16.1 (StataCorp. 2019. Stata Statistical Software: Release 16. College Station, TX, USA: StataCorp LLC).

## 3. Results

### 3.1. Group Numbers

In total, 45,140 patients fit our definition of polytrauma. Of these, 9918 had no recorded primary outcome (survival vs. non-survival), and a further 2877 were not recorded as having a comorbidity status. The characteristics of the excluded patients were investigated and were found to be similar to those in the study population (no selection bias was incurred by excluding them). More specifically, we considered median age, gender, ISS and presenting physiology.

The remaining 32,345 patients were categorised into either: Group 1—Diabetes present (*n* = 2271); Group 2—No diabetes but other known PMC (*n* = 16,319); or Group 3—no known PMC (*n* = 13,755) (Table 1). The diabetic cohort had a median age of 69.3 years, which was significantly older than the polytrauma patients with other/no comorbidities (median ages of 59.8 and 35.9 years, respectively), *p* < 0.001 (Table 1).

The prevalence of diabetes has increased in the UK since the original data were published. This increase can be seen in the new data, with a prevalence of 7% (2271/32,345) compared to 4% in the previous dataset [3].

### 3.2. Primary OutcomeI

All-cause mortality across the study population was 15.2%. This was once again shown to be statistically higher in the diabetic cohort compared to the other groups (21.3 vs. 17 vs. 11.5%), *p* < 0.0001 (Table 1). The overall hospital mortality rate compared to the 2003–2011 data was reduced for all three groups, but the highest magnitude reduction was seen in the diabetic group (11.1 vs. 3.1 vs. 1.4% reduction from the 2003–2011 rates).

### 3.3. General Observations

In this study population, 68.9% of the polytrauma patients were male and had an average age of 50.3 years. On arrival, the average ISS for each cohort was 29, their median GCS was 15 and they had a median number of one operation (Table 1).

Diabetic patients were shown to have higher systolic blood pressure (SBP) on arrival than the other two cohorts (137 vs. 131 vs. 126 mmHg), *p* < 0.001. However, the healthy cohort was shown to be more likely to be tachycardic on arrival (86 vs. 86 vs. 90 beats per minute), *p* < 0.001.

The number of days in the intensive care unit (ICU) was 5 for the diabetic and comorbid cohort compared to 4 days for the healthy cohort. However, the patients in the healthy cohort were more likely to need ICU admission (44 vs. 46.1 vs. 56.3%), *p* < 0.0001 (Table 1).

Diabetic patients were noted to be more likely to die later in their admission than the other cohorts, with a median number of hours to death of 105 compared to 80.3 in the comorbid group and 58.9 in the healthy cohort (*p* < 0.0001) (Table 1). Upon comparison of these figures against the original dataset, there is a clear shift observed, with individuals in all patient groups dying later in their admission. For the diabetic group, the time to death increased over two-fold (42.9 vs. 105), over three-fold in the comorbid group (23.3 vs. 80.3) and over five-fold in the healthy group (10.7 vs. 58.9) (Table 1).

### 3.4. Body Regions

All of the cohorts had similar rates of polytrauma associated with an AIS 3+ injuries of the following body regions: head (59.0 vs. 57.0 vs. 54.3%), *p* < 0.001, and extremity (35.7 vs. 34.3 vs. 33.9%). However, chest (77.0 vs. 78.2 vs. 83.9%), *p* < 0.001, and abdominal injuries (16.0 vs. 18.6 vs. 27.9), *p* < 0.001, were more frequently seen in the healthy cohort (Table 2).

### 3.5. Mechanism of Injury

The diabetic cohort was less likely to have suffered polytrauma as a result of a road traffic collision (RTC), stabbings/shootings or penetrating injuries than the other cohorts (Table 2). In addition, the diabetic group was considerably more likely to have sustained their polytrauma from lower-energy falls (<2 m) (38.9 vs. 28.5 vs. 5.3%), *p* < 0.0001.

### 3.6. Complications

As a result of the trends seen in the original data, further analysis has once again been undertaken to assess the percentage of patients that developed specific subsets of complications. These include thrombotic complications (acute respiratory distress syndrome, deep vein thrombosis and pulmonary embolism), infective complications (pneumonia, wound infection, sepsis and urinary tract infection) and organ failures (renal failure or myocardial infarction, arrhythmia and cardiac arrest). Each cohort had an equal to or greater than 95% chance of developing one of any of the above complications, with no single cohort being statistically more likely than another.

The diabetic cohort developed more infective complications compared to the other cohorts (12.9 vs. 10.5 vs. 5.3%), and unlike the trend seen in the original dataset, this difference has now been demonstrated to be statistically significant (*p* < 0.0001). In comparison to the original data (10.4 vs. 8.3 vs. 6.6%), the diabetic cohort has now been shown to be nearly 2.5 times more likely to develop infective complications compared to a patient in the previously healthy cohort (Table 3).

Similar to infective complications, renal failure has now been shown to be statistically more likely to develop in diabetic patients compared to the other cohorts (5.3 vs. 2.8 vs. 1.0), *p* < 0.0001 (Table 3). In comparison to the previous data, all cohorts were shown to have had an increased likelihood of developing renal failure, but the relatively low number of patients developing this complication in the original dataset means it would not be possible to show a statistically significant difference. Despite having the least apparent percentage increase in renal failure complications from the original dataset, the diabetic cohort has now been shown to be 2.4 times more likely to develop renal failure than the healthy cohort (Table 3).

Thrombotic complications have been dramatically reduced since the original data were presented, with an overall reduction across the study cohorts from 2.5% in the original dataset to just 0.3% in our dataset. With such low numbers of these complications, no statistical significance between the three cohorts has been shown in the data (0.4 vs. 0.4 vs. 0.3%). Regarding the diabetic cohort, the number of thrombotic complications has reduced 13-fold compared to the original data (5.4 vs. 0.4%) (Table 3).

All of the cohorts appear to have had a slight reduction in their likelihood of developing cardiac complications compared to the original dataset (3.6 vs. 3.1 vs. 1.7% current data; 5.9 vs. 4.4 vs. 2.1% old data). The difference between cohorts in the new dataset has not been shown to be statistically significant.

### 3.7. Mortality by Body Region, Complication, and Mechanism of Injury

The diabetic cohort was more likely to die from their polytrauma, comprising any given body region injured (Table 3). Most notable of these injury locations, the diabetic cohort was twice as likely to die after sustaining polytrauma to an extremity injury (AIS ≥ 3) than a patient in the healthy cohort (16.7 vs. 8.3%), *p* < 0.0001.

The diabetic cohort continues to have higher mortality from head injuries (25.4 vs. 22.2 vs. 17.8%), *p* < 0.0001. However, the same diabetic cohort has seen a dramatic reduction in mortality from polytrauma involving head injury compared to the original dataset, reduced from 43.9% to 25.4%. Diabetic patients were also more likely to die after developing an infective complication than any other cohort and 3.6 times more likely to die than a previously healthy person (26.9 vs. 20.8 vs. 7.4%), *p* < 0.05.

In addition, the diabetic cohort has been shown to be more likely to die after suffering polytrauma from an RTC (19.2 vs. 14.2 vs. 12.1%) and a fall from >2 m (25.7 vs. 19.7 vs. 10.5%), *p* < 0.0001, for both groups.

### 3.8. Mortality Prediction Model

Table 4 demonstrates the findings of a mortality prediction model. This shows that increasing ISS and age are associated with increasing mortality. It appears that the presence of diabetes, even when taking into consideration age, ISS and GCS, lead to an increase in the prediction of mortality, with an odds ratio (OR) of 1.36 (*p* < 0.0001).

## 4. Discussion

In comparison to the previous results, we can see that the study population included in the three cohorts over a similar length of time (2003–2011 vs. 2012–2019) has increased by over five-fold from 5489 to 32,345. This increase may be explained by numerous factors, including an increased level of trauma, better identification of trauma patients and an increase in the level of reporting to TARN. Furthermore, the reduced percentage of patients excluded in this dataset (28%) compared to the previous dataset (43%) is likely due to more accurately and more completely collated data from each hospital. In turn, this may suggest that the current dataset is of higher quality and, therefore, more likely to show reliable differences in outcome.

The UK population increased from 63.7 million to 66.8 million between 2012 and 2019 [9]. During the same time period, the prevalence of diabetes increased from 3 million to 4 million [7]. The original dataset comprised 4% diabetic patients, which has now risen to 7% in the new dataset. This figure would be slightly higher than the anticipated 5.8% expected in 2019 and certainly higher than the expected figure over the entire time of the dataset. One may attribute this finding to better primary care screening for diabetes. Of note, this rise to 7% was not reflected in the original dataset and could suggest that diabetes is an independent risk factor for suffering polytrauma/more severe trauma after sustaining an injury with any given level of energy. This has certainly been shown at low-energy levels in these results, with diabetic patients having an increased likelihood of sustaining polytrauma from lower-energy mechanisms (fall < 2 m) and at high-energy levels, with increased mortality from high-energy mechanisms (RTC and falls of >2 m). Since our original findings focused more on the microvascular changes associated with diabetes and the impact on developing complications, there has been an increase in evidence that prolonged diabetes has an impact on bone quality [10]. This may be a mechanism by which diabetic patients sustain polytrauma from low-energy mechanisms and die from the symptoms of high-energy mechanisms.

Interestingly, since the publication of our original paper [3], the understanding and treatment of diabetic patients suffering from trauma have been identified as having increased importance [11,12]. Evidence of this and the improvements seen by the introduction of the major trauma network [3] can be seen in the narrowing complication rate developing between the three cohorts. The likelihood of dying due to these complications is reduced too.

Herein lies the answer to the aim of this study, which is to highlight that the development of the major trauma network has affected the outcomes of diabetic patients. However, the improvement seen could also be related to the fact that diabetic patients are physiologically better in recent years compared to how they were previously (2003–2011), as there have been improvements in insulin schemes, monitoring, sensors and lifestyle change consultations [13,14].

We know that, in general, all patient outcomes have improved [3], but the new data suggest that diabetic patients are benefiting more than others. Despite this, these individuals do remain behind, and it may be that we are entering a stage where the judicious treatment of diabetic patients is no longer enough to reduce their risk and that other focus should be on primary management (reducing glycated haemoglobin—HbA1c) and reducing the prevalence of diabetes within the local population. One such programme is the “Healthier You” NHS Diabetes Prevention Programme (NHS DPP), which aims to reduce the number of diabetic patients in the UK and help some individuals into diabetes remission [7].

No more so is the impact seen on the mortality of patients with diabetes suffering polytrauma associated with head injury (AIS ≥ 3), with their mortality reducing from 43.9% to 25.4%.

The shift in the timing of mortality for all cohorts to later in their admissions is likely due to the improved treatment of massive haemorrhage through the use of tranexamic acid and massive transfusion pathways [15]. Within itself, this may imply that an increased time to death is more likely due to the complications developed. As discussed in the first study and now shown in this study, diabetic patients are more likely to develop certain complications and are also more likely to die from them.

Further benefits for all cohorts include the dramatic reduction in venous thromboembolic (VTE) events. This is likely due to the increased awareness of VTE and the routine use of VTE prophylaxis in trauma patients. The reduced rates of cardiac complications may be due to the improved use of blood products in resuscitation. Although the numbers involved in the original dataset were very low, it does appear that the rates of renal failure in trauma patients have increased. This may also be due to changes in initial fluid management, with a movement away from aggressive initial crystalloid-based fluid resuscitation [15].

As with the previous paper, this still remains a retrospective, observational, cohort study of prospectively collected data. The quality of the data used depends on the accuracy of the reporting hospitals. No interventions have been assessed, and all conclusions are observational only. The cohort with prior medical comorbidities has been used to provide, as near as possible, a matching cohort of patients to adjust for the diabetic population, who tend to be older than the average age of a patient suffering from polytrauma. No differentiation has been made between Type I and Type II diabetes mellitus, given that 90% of patients with diabetes suffer from Type II [7].

## 5. Conclusions

The prevalence of diabetes mellitus in polytrauma patients has increased since the first dataset, and diabetes remains an independent risk factor for mortality following polytrauma. In many of the areas analysed, there have been improvements in outcomes across all three cohorts and a reduction in the extent to which diabetic patients suffer from poorer outcomes. Schemes such as the “Healthier You” NHS DPP, which are aimed at reducing the prevalence of diabetes, may be the best way to improve outcomes in the future [7].

## Figures and Tables

**Table 1:** Characteristics of the polytrauma population in England and Wales using previous medical comorbidity (PMC) status, (SBP = Systolic Blood Pressure), (ISS = Injury Severity Score), GCS (Glasgow Comma Score).

	Diabetes I	Known PMC No Diabetes II	Definitely No PMC III	Total	*p*-Value	Post-Hoc	Not Recorded PMC
Total	2271	16,319	13,755	32,345			2877
Male	1442 (63.5%)	10,036 (61.5%)	10,795 (78.5%)	22,275 (68.9%)	<0.0001	I vs. II *p* = 0.081	1809 (62.9%)
Female	829 (36.5%)	6283 (38.5%)	2958 (21.5%)	10,070 (31.1%)			1068 (37.1%)
Age	69.3 (16.5)	59.8 (22.5)	35.9 (18.7)	50.3 (24.1)	<0.0001		41.7 (25.9–62.4)
ISS	29 (22–36)	29 (22–36)	29 (25–38)	29 (22–38)	<0.0001		34 (25–42)
GCS on arrival, median (IQR)	15 (14–15)	15 (14–15)	15 (14–15)	15 (14–15)	0.23		15 (7 –15)
SBP on arrival, median (IQR)	137 (116–159)	131 (113–151)	126 (110–142)	129 (112–148)	<0.0001		124 (104–143)
Pulse on arrival, median (IQR)	86 (74–101)	86 (73–101)	90 (75–108)	88 (74–104)	<0.0001	I vs. II *p* = 0.999	90 (74–110)
Days in hospital, median (IQR)	15 (7–31)	14 (7–27)	11 (6–23)	13 (6–26)	<0.0001		7 (1–19)
Hours to death, median (IQR)	105 (33.5–327.4)	80.3 (18.3–245.1)	27.9 (6–99.2)	58.9 (13.1–198)	<0.0001		6.6 (0.9–52.1)
Mortality, *n* (%)	484 (21.3%)	2831 (17.3%)	1588 (11.5%)	4903 (15.2%)	<0.0001		1156 (40.2%)
Number of operations, median (IQR)	1 (1–2)	1 (1–2)	1 (1–2)	1 (1–2)	<0.0001		1 (1–2)
Hours to operations, median (IQR)	23.9 (9.2–63.7)	20.6 (5.7–56.6)	15.6 (3.8–42.9)	18.4 (4.6–49.2)	<0.0001		11.4 (2.3–43.3)
Critical care stay, *n* (%)	999 (44%)	7518 (46.1%)	7747 (56.3%)	16,264 (50.3%)	<0.0001	I vs. II *p* = 0.095	1459 (50.7%)
Days in critical care, median (IQR)	5 (2–12)	5 (2–11)	4 (2–11)	4 (2–11)	<0.0001	I vs. II *p* = 0.152	7 (1–19)

**Table 2:** Polytrauma (Abbreviated Injury Score of 3+ ≥ 2 body regions), with known outcome. AIS (Abbreviated Injury Scale); ARDS (Adult Respiratory Distress Syndrome); DVT (Deep Vein Thrombosis); PE (Pulmonary Embolism); MI (Myocardial Infraction); RTC (Road Traffic Collision).

	Diabetes I	Known PMC No Diabetes II	Definitely No PMC III	Total	*p*-Value	Post-Hoc
Head AIS 3+, *n* (%)	1340 (59%)	9300 (57%)	7474 (54.3%)	18,114 (56%)	<0.0001	I vs. II *p* = 0.105
Chest AIS 3+, *n* (%)	1748 (77%)	12,761 (78.2%)	11,544 (83.9%)	26,053 (80.5%)	<0.0001	I vs. II *p* = 0.250
Abdomen AIS 3+, *n* (%)	363 (16%)	3030 (18.6%)	3844 (27.9%)	7237 (22.4%)	<0.0001	
Extremity AIS 3+, *n* (%)	810 (35.7%)	5590 (34.3%)	4662 (33.9%)	11,062 (34.2%)	0.393	
Any Complication, *n* (%)	699 (30.8%)	4440 (27.2%)	2259 (16.4%)	7398 (22.9%)	0.535	
ARDS, DVT, PE, *n* (%)	9 (0.4%)	60 (0.4%)	42 (0.3%)	111 (0.3%)	0.994	
Pneumonia, Wound Infection, Sepsis, Urinary tract infection, *n* (%)	294 (12.9%)	1717 (10.5%)	733 (5.3%)	2744 (8.5%)	<0.0001	
Renal failure, *n* (%)	120 (5.3%)	453 (2.8%)	140 (1%)	713 (2.2%)	<0.0001	
MI, Arrhythmia, Cardiac Arrest, *n* (%)	82 (3.6%)	509 (3.1%)	240 (1.7%)	831 (2.6%)	0.812	
RTC, *n* (%)	833 (36.7%)	6368 (39%)	9613 (69.9%)	16,814 (52%)	<0.0001	
Fall more > 2 m, *n* (%)	482 (21.2%)	4243 (26%)	2001 (14.5%)	6726 (20.8%)		
Fall < 2 m, *n* (%)	883 (38.9%)	4656 (28.5%)	723 (5.3%)	6262 (19.4%)		
Stabbing/Shooting, *n* (%)	18 (0.8%)	371 (2.3%)	649 (4.7%)	1038 (3.2%)		
Other, *n* (%)	55 (2.4%)	681 (4.2%)	769 (5.6%)	1505 (4.7%)		
Penetrating, *n* (%)	23 (1%)	411 (2.5%)	686 (5%)	1120 (3.5%)	<0.0001	

**Table 3:** Mortality by body region injury, complications incurred, and mechanism of injury. AIS (Abbreviated Injury Scale); ARDS (Adult Respiratory Distress Syndrome); DVT (Deep Vein Thrombosis); PE (Pulmonary Embolism); MI (Myocardial Infraction); RTC (Road Traffic Collision).

	Diabetes I			Known PMC No Diabetes II			Definitely No PMC III		
	Alive	Dead	*p*-Value	Alive	Dead	*p*-Value	Alive	Dead	*p*-Value
Head AIS 3+, *n* (%)	1000 (74.6%)	340 (25.4%)	<0.0001	7233 (77.8%)	2067 (22.2%)	<0.0001	6141 (82.2%)	1333 (17.8%)	<0.0001
Chest AIS 3+, *n* (%)	1356 (77.6%)	392 (22.4%)	0.018	10,422 (81.7%)	2339 (18.3%)	<0.0001	10,087 (87.4%)	1457 (12.6%)	<0.0001
Abdomen AIS 3+, *n* (%)	297 (81.8%)	66 (18.2%)	0.112	2593 (85.6%)	437 (14.4%)	<0.0001	3442 (89.5%)	402 (10.5%)	<0.0001
Extremity AIS 3+, *n* (%)	675 (83.3%)	135 (16.7%)	<0.0001	4821 (86.2%)	769 (13.8%)	<0.0001	4275 (91.7%)	387 (8.3%)	<0.0001
Any Complication, *n* (%)	488 (69.8%)	211 (30.2%)	0.195	3342 (75.3%)	1098 (24.7%)	0.02	1888 (83.6%)	371 (16.4%)	<0.0001
ARDS, DVT, PE, *n* (%)	6 (66.7%)	3 (33.3%)	0.378	46 (76.7%)	14 (23.3%)	0.220	36 (85.7%)	6 (14.3%)	0.346
Pneumonia, Wound Infection, Sepsis, urinary tract infection, *n* (%)	215 (73.1%)	79 (26.9%)	0.013	1360 (79.2%)	357 (20.8%)	<0.0001	679 (92.6%)	54 (7.4%)	<0.0001
Renal failure, *n* (%)	78 (65%)	42 (35%)	<0.0001	329 (72.6%)	124 (27.4%)	<0.0001	109 (77.9%)	31 (22.1%)	0.072
MI, Arrhythmia, Cardiac Arrest, *n* (%)	27 (32.9%)	55 (67.1%)	<0.0001	174 (34.2%)	335 (65.8%)	<0.0001	73 (30.4%)	167 (69.6%)	0.56
RTC, *n* (%)	673 (80.8%)	160 (19.2%)		5465 (85.8%)	903 (14.2%)		8454 (87.9%)	1159 (12.1%)	<0.0001
Fall more > 2 m, *n* (%)	358 (74.3%)	124 (25.7%)		3408 (80.3%)	835 (19.7%)		1790 (89.5%)	211 (10.5%)	<0.0001
Fall < 2 m, *n* (%)	689 (78%)	194 (22%)	0.004	3669 (78.8%)	987 (21.2%)	<0.0001	649 (89.8%)	74 (10.2%)	<0.0001
Stabbing/Shooting, *n* (%)	17 (94.4%)	1 (5.6%)		336 (90.6%)	35 (9.4%)		592 (91.2%)	57 (8.8%)	<0.0001
Other, *n* (%)	50 (90.9%)	5 (9.1%)		610 (89.6%)	71 (10.4%)		682 (88.7%)	87 (11.3%)	0.941
Penetrating, *n* (%)	21 (91.3%)	2 (8.7%)	0.138	371 (90.3%)	40 (9.7%)	<0.0001	623 (90.8%)	63 (9.2%)	0.952

**Table 4:** Mortality Prediction Model. CI (confidence interval); GCS (Glasgow Coma Score); ISS (Injury Severity Score); PMC (pre-existing medical condition).

Variables	Odds Ratio	*p*-Value	95% CI	
ISS	1.04	<0.0001	1.03	1.04
GCS	0.72	<0.0001	0.72	0.73
Age	1.05	<0.0001	1.05	1.05
Sex (Female)	1.11	0.373	0.88	1.41
Sex by Age interaction	1.00	0.459	1.00	1.00
No PMC (reference)	1			
Diabetes	1.36	<0.0001	1.16	1.58
PMC and no diabetes	1.12	0.025	1.01	1.23

## Data Availability

The data presented in this study are available upon reasonable request from the corresponding author (PVG).
